# Cost, Usability, Credibility, Fairness, Accountability, Transparency, and Explainability Framework for Safe and Effective Large Language Models in Medical Education: Narrative Review and Qualitative Study

**DOI:** 10.2196/51834

**Published:** 2024-04-23

**Authors:** Majdi Quttainah, Vinaytosh Mishra, Somayya Madakam, Yotam Lurie, Shlomo Mark

**Affiliations:** 1 College of Business Administration Kuwait University Kuwait Kuwait; 2 College of Healthcare Management and Economics Gulf Medical University Ajman United Arab Emirates; 3 Information Technology Birla Institute of Management Technology Knowledge Park - II Greater Noida India; 4 Department of Management Ben-Gurion University Negev Israel; 5 Department of Software Engineering Shamoon College of Engineering Ashdod Israel

**Keywords:** large language model, LLM, ChatGPT, CUC-FATE framework, cost, usability, credibility, fairness, accountability, transparency, and explainability, analytical hierarchy process, AHP, total interpretive structural modeling, TISM, medical education, adoption, guideline, development, health care, chat generative pretrained transformer, generative language model tool, user, innovation, data generation, narrative review, health care professional

## Abstract

**Background:**

The world has witnessed increased adoption of large language models (LLMs) in the last year. Although the products developed using LLMs have the potential to solve accessibility and efficiency problems in health care, there is a lack of available guidelines for developing LLMs for health care, especially for medical education.

**Objective:**

The aim of this study was to identify and prioritize the enablers for developing successful LLMs for medical education. We further evaluated the relationships among these identified enablers.

**Methods:**

A narrative review of the extant literature was first performed to identify the key enablers for LLM development. We additionally gathered the opinions of LLM users to determine the relative importance of these enablers using an analytical hierarchy process (AHP), which is a multicriteria decision-making method. Further, total interpretive structural modeling (TISM) was used to analyze the perspectives of product developers and ascertain the relationships and hierarchy among these enablers. Finally, the cross-impact matrix-based multiplication applied to a classification (MICMAC) approach was used to determine the relative driving and dependence powers of these enablers. A nonprobabilistic purposive sampling approach was used for recruitment of focus groups.

**Results:**

The AHP demonstrated that the most important enabler for LLMs was *credibility*, with a priority weight of 0.37, followed by *accountability* (0.27642) and *fairness* (0.10572). In contrast, *usability*, with a priority weight of 0.04, showed negligible importance. The results of TISM concurred with the findings of the AHP. The only striking difference between expert perspectives and user preference evaluation was that the product developers indicated that *cost* has the least importance as a potential enabler. The MICMAC analysis suggested that cost has a strong influence on other enablers. The inputs of the focus group were found to be reliable, with a consistency ratio less than 0.1 (0.084).

**Conclusions:**

This study is the first to identify, prioritize, and analyze the relationships of enablers of effective LLMs for medical education. Based on the results of this study, we developed a comprehendible prescriptive framework, named CUC-FATE (Cost, Usability, Credibility, Fairness, Accountability, Transparency, and Explainability), for evaluating the enablers of LLMs in medical education. The study findings are useful for health care professionals, health technology experts, medical technology regulators, and policy makers.

## Introduction

### Background

Natural language programming solutions have been available for the last 15 years. However, these models recently witnessed an avalanche breakdown with the launch of ChatGPT by OpenAI, a company that was only established recently (December 2015) after receiving an investment from Elon Musk and others. ChatGPT is a generative language model tool that enables users to converse with machines about various subjects. With 1.6 billion monthly users, this freemium is the fastest-growing application in the history of the internet. Since its release on November 30, 2022, ChatGPT has sparked much discussion and enthusiasm in multiple industries, including medicine. ChatGPT and related technologies have been identified as disruptive innovations with the potential to revolutionize academia and scholarly publishing [1]. Additionally, preliminary research suggests that ChatGPT has practical applications throughout the clinical workflow [2].

The introduction of ChatGPT and the subsequent release of several extended products and functional plugins have profoundly impacted scientific researchers. These products have also influenced the ideas and methodologies used in traditional research, including recommendation, emotion recognition, and information generation. ChatGPT’s assistance has improved some of the associated work in these fields, particularly with providing helpful supplementary information to raise the caliber of data generation. With the integration of machine learning and artificial intelligence (AI) technologies, medical imaging has advanced quickly. Among these developments, using cutting-edge language models such as large language models (LLMs), ChatGPT, and GPT-4 has shown significant promise in elevating several elements of medical imaging and revolutionizing radiology. These models can produce and comprehend human-like text owing to access to various textbooks, journals, and research materials available on the internet. This could provide the necessary context and prior knowledge to support a variety of tasks involving medical imaging, such as synthesis, reconstruction, analysis, segmentation, interpretation, automated reporting, and more. These technologies have further been improved using supervised and reinforcement learning methods based on OpenAI’s GPT LLMs. These models have shown excellent performance in various natural language processing (NLP) tasks, including language translation, text summarization, and question-answering. The models have been pretrained on enormous amounts of text data. Users can ask questions, obtain responses, and engage in genuine conversation with the bot given ChatGPT’s human-like conversational experience.

ChatGPT and other LLMs remain a research hotspot in multimedia analysis and application. However, several crucial difficulties must be resolved, including (1) improving interactions with ChatGPT to collect more useful auxiliary information, (2) methods to combine ChatGPT with traditional inquiries to fully exploit its benefits, and (3) analyzing the data obtained from ChatGPT for their incorporation with the intended usage. A particularly significant challenge is to effectively use past information obtained with such huge models and to ensure consistency and complementary features across many modalities to improve multimodal generation performance, which is especially relevant for AI-generated content. The finest use cases for ChatGPT, a well-liked chatbot built on a potent AI language model, are still being worked out. ChatGPT can provide help in writing an essay, thesis, or dissertation by creating a research question, developing a plan, developing literary concepts, rewriting text, and getting feedback. Moreover, the NLP and automated data analysis capabilities offered by ChatGPT enable researchers, marketers, and organizations to analyze text quickly and accurately. Via its AI-powered functions, ChatGPT can help to spot significant trends and insights in a data set that might otherwise be challenging to find. Additionally, ChatGPT can assist with the creation of top-notch prompts for paper analysis.

### LLM Functionality

ChatGPT is a prediction system that anticipates what it should write based on previously processed texts. This type of AI is known as a language model. However, ChatGPT offers more promise than its predecessors given that it is trained on enormous amounts of data, with the majority of these data originating from the abundant supply of data available on the internet. According to OpenAI, ChatGPT was also trained on examples of back-and-forth human interaction, which results in a conversation style that is much more human than that of other chatbots, thus advancing the capability of NLP solutions.

NLP is a field of AI employing linguistics, statistics, and machine learning to enable computers to comprehend spoken language. NLP systems can infer meaning from spoken or written words, including all of the subtleties and complexities of an accurate narrative text. This makes it possible for machines to obtain value from even unstructured data. NLP has witnessed significant advancements in recent years. An LLM is a deep-learning algorithm that can be used to perform NLP tasks, including, among other abilities, summarizing and generating text. As one of the main applications, LLM-based chatbots are computer programs that can simulate conversations with human users. NLP techniques can be used to enable chatbots to understand and respond to user input. LLM uses deep-learning techniques to understand and generate human language, which requires training on vast amounts of text data and then uses statistical algorithms to learn patterns and relationships within language. These models can perform various tasks, including language translation, question-answering, sentiment analysis, and summarization. With ChatGPT, users can learn, compare, and validate answers for different academic subjects, including physics, math, and chemistry, as well as abstract topics such as philosophy and religion [3]. Users can also generate human-like text such as news articles, chatbot conversations, and even literary works such as essays and romantic poems. The main difference of GPTs from other LLMs lies in their architecture and training methodology. GPTs are based on a deep-learning architecture known as a “transformer.” Transformers are designed to process sequential data such as language more efficiently than other architectures. LLMs are currently at the forefront of intertwining AI systems with human communication and everyday life [4]. Large pretrained language models have significantly advanced NLP research with respect to various applications [5,6]. Although these more complicated language models can produce complex and coherent natural language, several recent studies have shown that they can also pick up unfavorable social biases that can feed into negative stereotypes [7].

### NLP in Health Care

Health care consumers may turn to the research literature for information not provided in patient-friendly documents. However, reading medical literature can be difficult. One study identified four key elements made possible by NLP to increase access to medical papers: explanations of foreign terminology, plain language section summaries, a list of crucial questions that direct readers to the portions that provide the answers, and simple language summaries of those passages [8]. Significant advancements in smart health care have been made in recent years, with new AI technologies enabling a range of intelligent applications in various health care contexts. NLP, as a fundamental AI-powered technology that can analyze and comprehend human language, is crucial for smart health care [9]. NLP methods have been utilized to organize data in health care systems by sifting out pertinent information from narrative texts to offer information for decision-making. Thus, NLP approaches help to lower health care costs and are essential for streamlining health care procedures [10]. Advancements in NLP will make robotic process automation possible in health care, which can further drive efficiency. Health care data are complex, which should be given due consideration at the time of designing health care applications. Deep-learning approaches such as convolutional neural network and recurrent neural network models have become prominent in health care applications, demonstrating promising accuracy. Nevertheless, there is still substantial room for improvement of these models to enable their usage without human supervision. Deep-learning techniques offer an effective and efficient model for data analysis by revealing hidden patterns and extracting valuable information from a large volume of health data, which standard analytics cannot perform within a given time frame [11].

### ChatGPT in Medical Education

ChatGPT has many potential applications in health care education, research, and practice [12], which can enhance medical education by helping students develop subjective learning and expression skills [13]. The number of ChatGPT users has shown exponential growth and the tool is increasingly utilized by students, residents, and attending physicians to direct learning and answer clinical questions [14]. However, authors using ChatGPT professionally for academic work should exercise caution as it remains unclear how ChatGPT handles hazardous content, false information, or plagiarism [15]. While ChatGPT can simplify the task of radiological reporting, there is still a chance of inaccurate statements and missing medical information [15]. Therefore, the tool needs refinement before it can be used widely with confidence in medicine [16]. A recent review explored ChatGPT’s applications and reported various challenges such as ethical concerns, data biases, and safety issues [17]. Thus, it is imperative to balance AI-assisted innovation and human expertise [18]. ChatGPT has quickly gained significant attention from academia, research, and industries despite these shortcomings. The first aim of this study was therefore to determine the requirements, or enablers, for a successful LLM application in medical education using a narrative review of the existing literature.

### Enablers of LLM for Medical Education

For the purpose of this study, we refer to enablers as the factors, resources, or conditions that facilitate or support achieving a good LLM application for medical education. Medical education prepares would-be physicians and other health care professionals with the knowledge, skills, and attitudes necessary for competent and compassionate patient care. The general definition of an enabler is a factor that makes it easier for a goal to be realized or for someone to accomplish a particular task. Enablers of LLM for medical education can be tangible or intangible and should play a crucial role in achieving the outcomes expected from the application.

As LLMs are trained on massive data, they are resource-demanding tools. Therefore, the cost of training an LLM for medical education may be prohibitive [19]. Accordingly, it is imperative to use efficient computing to address this issue [20]. Usability is one of the key criteria that determines the usefulness of an application in medical education, and LLMs are no exception [21]. The extant literature has highlighted usability as an important criterion for the successful implementation of a new technology in education [22]. Similarly, the credibility of an application is another very important factor for technological interventions used in medical education [23,24]. Although ChatGPT has disclaimers about the source of information provided, it does not disclose its sources categorically, and can sometimes hallucinate about the source, which may be misleading to the user. LLMs also have reported issues with fairness, computation, and privacy. By perpetuating social prejudices and stereotypes, they risk causing unfair discrimination and physical harm, along with potential harm to the user’s reputation [25]. Ma et al [26] provided an overview of fairness of LLMs in multilingual and non-English situations, emphasizing the limitations of recent studies and the challenges faced by English-only methodologies [26].

Another issue of LLMs such as ChatGPT is related to their accountability, generally defined as taking responsibility for one’s obligation to treat others honestly and morally. However, it is unclear who will be held accountable and responsible if the LLM provides incorrect recommendations or forecasts for a particular downstream activity. Overall, employing LLMs is associated with considerable risk; therefore, precautions must be taken to minimize these risks and ensure their ethical and responsible use. To foster a cross-disciplinary global inclusive consensus on the ethical use, disclosure, and proper reporting of generative AI models such as GPT and other LLM technologies in academia, Cacciamani et al [23] proposed the ChatGPT, Generative Artificial Intelligence, and Natural Large Language Models for Accountable Reporting and Use Guidelines initiative in 2023. However, the underlying model of GPT3.5 deviates from the ethical guidelines proposed by Cacciamani et al [23]. Another important criterion reported for the medical applications of LLMs is transparency, which is an essential ethical consideration in the fields of science, engineering, business, and the humanities. Transparency refers to functioning in a way that makes it simple for others to observe what actions have been taken [27], thus representing a sign of responsibility, honesty, and openness. Conversely, LLMs are opaque to users. Recently suggested explainability techniques aim to make LLMs more transparent. Although these techniques are not a cure-all, they might form the basis for the development of models with fewer flaws or, at the very least, the ability to explain their logic. In their systematic experiments with synthetic data, Wu et al [28] demonstrated that autoregressive and masked language models can successfully learn to emulate semantic relations between expressions with strong transparency, where all expressions have context-independent denotations.

Finally, the LLMs used in medical education must be explainable, and the best freely available options lag in this respect. Most LLMs are complex models built using deep learning [29]; therefore, these models can produce better predictions with more information or network parameters, which comes at a cost of sacrificing explainability. Some models fail to describe how they came to their conclusion. Recently suggested explainability techniques aim to make language models more transparent. Even though these are not complete solutions, they can act as the basis for the development of less problematic models or, at the very least, models that can explain their logic. However, Du et al [30] identified false patterns detected by LLMs using explainability in their study.

### Need for This Study

The need for this study arises from the rapid integration of LLMs such as ChatGPT in various fields, including medical education. Although LLMs offer promising benefits for health care, their effective integration in medical education remains a developing area. Accordingly, the aim of this study was to identify and prioritize the key enablers for successful LLM implementation in medical education. This can in turn help to address the lack of comprehensive frameworks guiding the development and use of LLMs in this field. By exploring the dynamics of various enablers such as credibility, accountability, fairness, cost, usability, transparency, and explainability, this study provides a structured approach to enhance the quality and effectiveness of LLMs in educating health care professionals.

Specifically, this study was based on the following three major research questions: (1) What are the enablers of a suitable LLM application for medical education? (2) What is the relative importance of these enablers in achieving the goals of medical education? and (3) What is an approach to developing an LLM to achieve medical education goals? With this background, the following research objectives were set: (1) identify the enablers of a suitable LLM for medical education, (2) prioritize the identified enablers in achieving the goals of medical education, and (3) propose a framework for developing an LLM to achieve the medical education goals.

## Methods

### Study Design

To achieve the first research objective, we performed a narrative review of the extant literature published on technology solutions in medical education. A narrative review is a scholarly article synthesizing existing research on a particular topic in a narrative or story-like manner. Unlike systematic reviews or meta-analyses, which use rigorous methodologies to analyze and summarize research findings quantitatively, narrative reviews provide a qualitative, comprehensive overview of a subject. Narrative reviews often involve critical analysis and discussion, integrating the authors’ expertise and interpretation. Narrative reviews are thus useful for obtaining a broad understanding of a topic and identifying trends, gaps, and controversies within a field.

Two authors (SM and VM) searched the Scopus, Web of Science, and Google Scholar databases to identify suitable literature for our narrative review. The inclusion criteria were articles published in the English language in the last 5 years. In the second stage, duplicates and articles for which the full text was unavailable were eliminated. The identified enablers from this review were then used to address the first research question. These enablers were presented in front of a focus group comprising seven experts working in universities and institutions delivering medical education in India and the United Arab Emirates to validate the selection (Table 1). The focus group endorsed the choice of the enablers for further research; in addition, one article published in 2010 was added on the recommendation of the focus group as it was found to be useful in explaining competing interests in medical education. One author (VM) facilitated the focus group discussion to obtain the finalize list of enablers.

**Table 1:** Characteristics of the focus group for validation of identified enablers.

Expert	Qualification	Experience (years)	Age (years)	Nationality
Cardiologist	Masters in Medicine	12	42	India
Endocrinologist	Masters in Medicine	20	45	India
Technology expert	Doctor of Philosophy	15	50	United Arab Emirates
Dentistry educator	Masters in Dentistry	10	40	United Arab Emirates
Podiatrist educator	Doctor of Philosophy	10	35	United Arab Emirates
Diabetes educator	Doctor of Philosophy	18	43	India
Nursing educator	Doctor of Philosophy	15	41	United Arab Emirates
Radiologist	Doctor of Philosophy	12	41	India

### Analytical Hierarchy Process Modeling

An analytical hierarchy process (AHP) was utilized to achieve the second study objective of prioritizing the identified enablers for developing an LLM for medical education. The AHP is a popular method for determining the relative importance of the criteria in a multicriteria decision analysis task. To date, the AHP has been extensively used in the management and social science fields [31]. The advantage of this process is that it incorporates the mechanisms to assure reliability in the decision-making case of ambiguity. Some researchers have suggested using a “fuzzy” version of the AHP [32] and others have suggested using the entropy weight method to reduce the negative effect of individual subjective evaluation bias on the accuracy of comprehensive evaluation [33]. Since the ranking obtained by the AHP method was further validated by total interpretive structural modeling (TISM) in this study (see below), fuzzy logic or entropy weight was avoided in our AHP modeling. The five steps used for AHP are: (1) defining the decision problem, (2) creating a hierarchy, (3) pairwise comparison, (4) deriving a weighted priority, and (5) consistency check for decision. We used the Delphi method for pairwise comparisons. A cut-off value of 75% was used to accept the value for the pairwise comparison. The standard scale proposed by Saaty [34] was used for the pairwise comparison.

### TISM and Focus Groups

Finally, to address the third research objective, we investigated the relationships among key enablers to inform the development of a suitable medical education LLM. A qualitative research design is useful to understand a phenomenon under study rather than assessing the strength and direction of causal relationships in a conceptual model [35]. For this purpose, we established a focus group with five experts in the fields of information technology and product development with relevant research experience. The details of this expert group are provided in Table 2.

**Figure 1:**
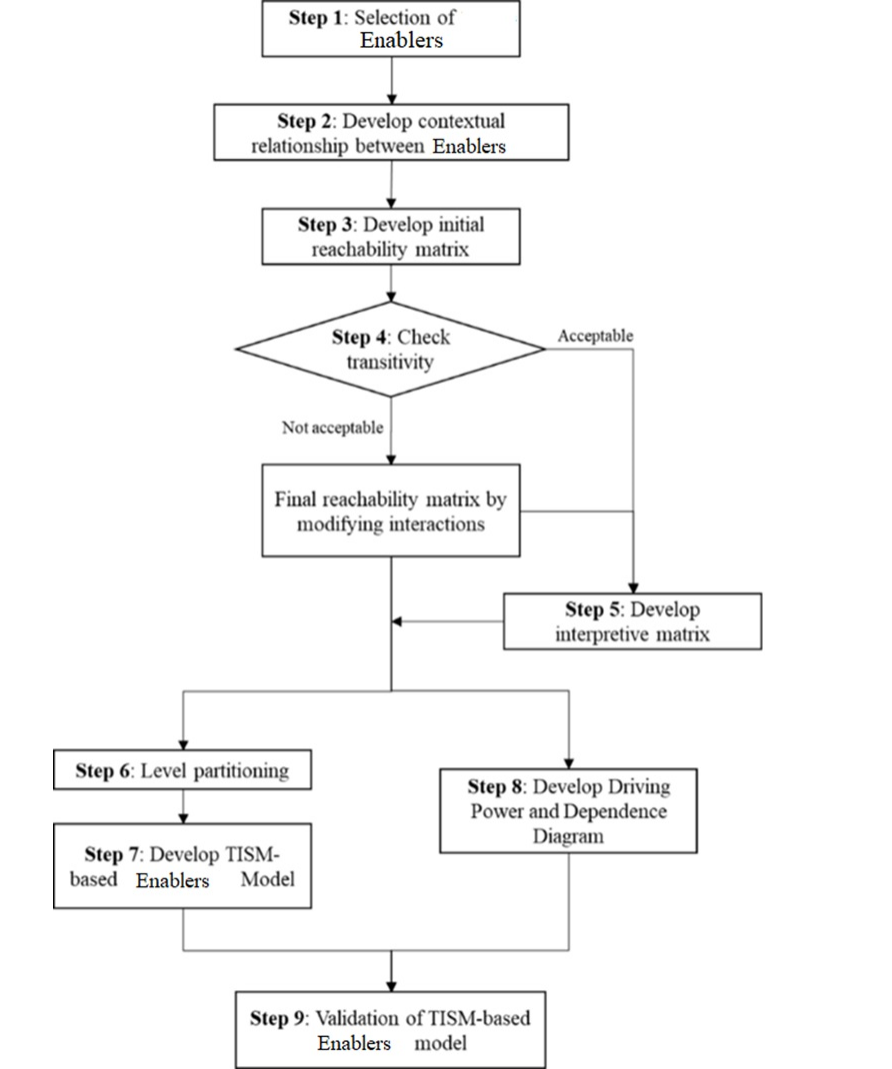
Summary of the total interpretive structural modeling (TISM) approach used in the study. Adapted from Mishra and Rana [33].

According to the information obtained from the focus group, TISM was used to model the enablers for a medical education LLM application. In his seminal paper, Sushil [36] provides a detailed account of the interpretation of interpretive structural modeling and TISM, highlighting the advantage of the latter over the former. For the sake of brevity, we have not included the details of the TISM method herein, which can be found in the relevant literature [37]. In brief, TISM is a process that converts poorly articulated mental models of systems into visible and well-defined models that are useful for gaining better understanding and decision-making. The presence and absence of a relationship between enablers were ascertained based on an unstructured interview of the focus group conducted by one researcher (SM). If more than 50% of the focus group members indicated that there is a relationship between two enablers, the enabler was considered to be present, which was coded as “Y.” An overview of the TISM approach used in this study is provided in Figure 1.

**Table 2:** Characteristics of the focus group used for total interpretive structural modeling.

Expert	Qualification	Experience (years)	Age (years)	Country
Product development	Masters in management	21	42	Singapore
Product development	Bachelors in engineering	21	42	United Arab Emirates
Technology expert	Bachelors in engineering	19	40	India
Technology expert	Masters in engineering	10	33	India
Decision science expert	Doctor of Philosophy	10	38	India

We further used cross-impact matrix multiplication applied to classification (MICMAC) analysis to evaluate the direct and indirect relationships among various elements in a complex system. MICMAC analysis is applied to the reachability matrix to classify the elements into four categories based on their driving power (ability to influence other elements) and dependence (level of being influenced by other elements).

### Ethical Considerations

This study, involving a qualitative focus group discussion, did not require approval from an ethical review board as it did not involve human subjects in a manner necessitating such review. No informed consent was required for the same reason. However, to maintain ethical standards, we ensured that all data collected were either anonymized or deidentified. This means that any information that could potentially identify individual participants was removed or altered to protect their privacy. No compensation was provided to participants, as is common in studies of this nature. This decision was made considering the study design and the ethical imperative to avoid undue influence on participants’ responses. The absence of compensation was communicated to all participants. Throughout the study, we adhered to strict data protection protocols to safeguard the confidentiality of the information shared during the focus group discussions. These measures included secure data storage, restricted access to authorized personnel, and adherence to data protection laws and regulations. This approach ensured that the privacy and integrity of participant information were always maintained.

## Results

### AHP Modeling

Based on the selected enablers identified for developing a suitable LLM medical education application according to the narrative review of the literature (Table 3), the focus group was asked to provide their input for pairwise comparison, and the resultant matrix [A] is presented in Table 4.

Once the initial comparison matrix was determined, the matrix was normalized and an average of each row was taken to calculate the priority weight [X]. The normalized matrix, priority weight, and rank of the enablers are given in Table 5. The priority weight, as the eigenvector, was further used to calculate the consistency ratio (CR).

**Table 3:** Summary of reported enablers of large language models for medical education.

Enabler code	Enabler	Description	References
E1	Cost	Cost of computation, including hardware, software, and energy requirement	[19,20]
E2	Usability	User-centric design, ease of use, and positive user experiences	[21,22]
E3	Credibility	Level of trust and reliability that users place in the application	[23,24]
E4	Fairness	Absence of unfair discrimination, physical harm, and harm to user reputation	[25,26]
E5	Accountability	Taking responsibility for the obligation to treat users with honesty and morality	[27,38]
E6	Transparency	Functioning in a way that makes it simple for others to observe what actions are taken	[27,30]
E7	Explainability	Ability to describe how the models came to their conclusion	[29,30]

**Table 4:** Initial pairwise comparison matrix for the analytical hierarchy process.a

Enablers	Cost (E1)	Usability (E2)	Credibility (E3)	Fairness (E4)	Accountability (E5)	Transparency (E6)	Explainability (E7)
E1	1	3	0.2	1	0.2	3	3
E2	0.33	1	0.11	0.33	0.11	1	1
E3	5	9	1	5	5	3	3
E4	1	3	0.2	1	0.2	3	3
E5	5	9	0.2	5	1	5	5
E6	0.33	1	0.33	0.33	0.2	1	1
E7	0.33	1	0.33	0.33	0.2	0.2	1

^a^Numbers represent the pairwise comparison of different enablers using the scale developed by Saaty [34].

**Table 5:** Normalized matrix and priority weight of enablers.

Enablers	Cost (E1)	Usability (E2)	Credibility (E3)	Fairness (E4)	Accountability (E5)	Transparency (E6)	Explainability (E7)	Priority weight	Rank
E1	0.077	0.1111	0.0844	0.077	0.0289	0.1852	0.1765	0.10572	3
E2	0.0254	0.037	0.0464	0.026	0.0159	0.0617	0.0588	0.03871	7
E3	0.3849	0.3333	0.4219	0.385	0.7236	0.1852	0.1765	0.37289	1
E4	0.077	0.1111	0.0844	0.077	0.0289	0.1852	0.1765	0.10572	3
E5	0.3849	0.3333	0.0844	0.385	0.1447	0.3086	0.2941	0.27642	2
E6	0.0254	0.037	0.1392	0.025	0.0289	0.0617	0.0588	0.0538	5
E7	0.0254	0.037	0.1392	0.025	0.0289	0.0123	0.0588	0.04674	6

Based on this matrix, the eigenvector X was calculated according to the following equation:


[A] X = *λ_max_* X – (1)

Using the data in Tables 4 and 5, *λ_max_* was obtained as follows:


[A]X = [0.76, 0.28, 3.46, 0.76, 2.26, 0.39, 0.34] – (2)

*λ_max_* = average {0.76/0.11, 0.24/0.04, 3.46/0.37, 0.76/0.11, 0.39/0.05, 0.34/0.05} – (3)

*λ_max_* = 7.66 – (4)

The consistency index (CI) was then calculated based on the *λ_max_* as follows: CI = (7.66 – 7)/6 = 0.11 – (5). Finally, the CR of the judgment was calculated by dividing the CI by the random index (RI). The RI value for a 7×7 matrix is 1.32 from the RI table. Thus, the CR becomes 0.084; as this is less than 0.1, it is considered to be acceptable.

### Modeling Relationships Among Enablers

We further used TISM for ascertaining the relationships among these seven enablers. Table 6 shows a matrix indicating the interrelationships between the enablers listed in Table 3, with “Y” indicating the existence of a relationship and “N” indicating no relationship. The resultant matrix is referred to as the structural self-interaction matrix.

In the next step, we replaced all “Ys” with 1s and all “Ns” with 0s and incorporated the transitivity rule to obtain the final reachability matrix shown in Table 7.

The next step in developing LLMs for medical education involved listing reachability and antecedent sets for each enabler, followed by level partitioning, which is an iterative process of assigning enablers at different levels. Enablers with similar intersection sets as reachability sets are placed at the top level. The process is then repeated until levels are established for all enablers. In this study, all enablers were assigned after three iterations; hence, there are three levels in the hierarchy. The summary of level partitioning is provided in Table 8. The level of an enabler is a reflection of its driving power and dependence power, as indicated in Table 7. The higher the level of the enabler, the more dependent it is, whereas the driving ability improves when moving to lower levels.

**Figure 2:**
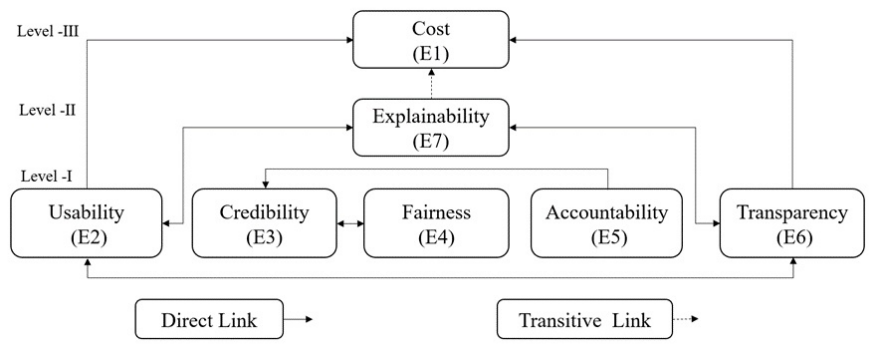
Diagraph of the total interpretive structural model for the development of large language models in medical education.

Once the level partitioning was complete, the TISM was developed and presented to the focus group for validation. Only significant transitive links were included in the model to facilitate interpretation. The final digraph for the TISM developed in the study is depicted in Figure 2.

**Table 6:** Structural self-interaction matrix for the identified enablers of large language models for medical education.

Enablers	Cost (E1)	Usability (E2)	Credibility (E3)	Fairness (E4)	Accountability (E5)	Transparency (E6)	Explainability (E7)
E1	Y^a^	Y	N^b^	N	N	Y	N
E2	Y	Y	N	N	N	Y	Y
E3	N	N	Y	Y	Y	N	N
E4	N	N	Y	Y	N	N	N
E5	N	N	Y	N	Y	N	N
E6	Y	Y	N	N	N	Y	Y
E7	N	Y	N	N	N	Y	Y

^a^Y: existence of a relationship between two enablers.

^b^N: no relationship exists between two enablers.

**Table 7:** Final reachability matrix of the enablers for developing large language models in medical education.

Enablers	Cost (E1)	Usability (E2)	Credibility (E3)	Fairness (E4)	Accountability (E5)	Transparency (E6)	Explainability (E7)	Driving power
E1	1	1	0	0	0	1	1	4
E2	1	1	0	0	0	1	1	4
E3	0	0	1	1	1	0	0	3
E4	0	0	1	1	0	0	0	2
E5	0	0	1	0	1	0	0	2
E6	1	1	0	0	0	1	1	4
E7	0	1	0	0	0	1	1	3
Dependence power	3	4	3	2	2	4	4	Not applicable

**Table 8:** Summary of label partitioning iterations (1 to 6).

Enablers, (Mi)	Reachability set, R(Mi)	Antecedent set, A(Ni)	Intersection set, R(Mi)∩A(Ni)	Level
1	1	1	1	III
2	1, 2, 6, 7	1, 2, 6, 7	1, 2, 6, 7	I
3	3, 4, 5	3, 4, 5	3, 4, 5	I
4	3, 4	3, 4	3, 4	I
5	3, 5	3, 5	3, 5	I
6	1, 2, 6, 7	1, 2, 6, 7	1, 2, 6, 7	I
7	7	1, 7	7	II

### Validation Analysis

**Figure 3:**
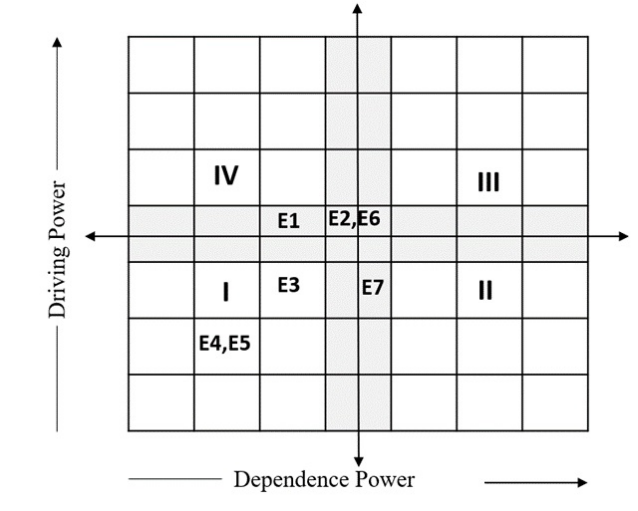
Cross-impact matrix-based multiplication applied to a classification (MICMAC) analysis for enablers of a large language model in medical education. I-IV indicate different levels of the enablers E1-E7. E1: cost; E2: usability; E3: credibility; E4: fairness; E5: accountability; E6: transparency; E7: explainability.

We further used MICMAC analysis to validate the study findings and derive conclusions. MICMAC analysis involves the development of a graph that classifies enablers based on their driving and dependence power. As shown in Figure 3, the first quadrant contains autonomous enablers E3 (Credibility), E4 (Fairness), and E6 (Accountability), indicating that the variables falling in this quadrant have low driving and dependence powers. The two enablers falling in the grey region between the third (linkage) and fourth (independent) quadrants are E2 (Usability) and E6 (Transparency), which have medium driving and dependence powers. Similarly, E7 (Explainability) falls in the grey region between the first (autonomous) and second (dependent) variables. Finally, E1 (Cost) falls under the fourth (independent) quadrant.

## Discussion

### Principal Findings

The results of the AHP suggested that credibility, followed by accountability are the foremost enablers for effective LLMs in medical education. The extant literature supports this finding, in highlighting the relevance of the source of information based on which the response was generated [39]. Similarly, the importance of defining accountability has been emphasized in the recent literature. For example, Tan et al [40] advocate for accountability as an important factor in increasing the adoption of LLMs in medical education, training, and practice. The next most important factors to consider are ethical issues such as fairness and cost. LLMs have been criticized for bias against gender or ethnic groups [17]. These problems need to be addressed to make LLMs effective in medical education. Moreover, training LLMs on billions of parameters is demanding; thus, only technology giants will launch these LLMs [41]. Governments should therefore ensure that the cost of using these LLMs does not become prohibitive for end users, who may resort to insufficient solutions that could ultimately affect the safety of patients.

In contrast to existing studies, transparency and explainability ranked fifth and sixth in importance in our analysis [40]. Many best practices related to health technology suggest that models should use explainable AI in medical devices [17]. The low priority of these enablers identified in this study indicates that the end user is unaware of the criticality of these factors; thus, health care professionals need to be educated about these issues as they are not technology savvy [42]. Governments should also establish guidelines for the approval of Software as Medical Devices so that these enablers are taken care of at the product development stage. Finally, the focus group indicated that usability is the least important factor among the seven enablers discussed. Although general-purpose LLMs such as ChatGPT are less cluttered, their performance is input-dependent. Improving the prompt use of the recommendation system can enhance the usability and accuracy of LLMs in medical education [43]. The expert group advised that the LLMs will improve on these factors with time.

The results from TISM suggested a slight difference in the perspective of product developers and end users, as the experts gave equal importance to the enablers credibility, fairness, accountability, transparency, and explainability. These results are consistent with extant literature published in peer-reviewed journals [40,41], as these are all features related to model development and training.

In contrast to earlier studies, the product developers and technology experts placed less significance on usability as an enabler, which was given a medium level [43]. Thus, the finding of the TISM validates the results of the AHP. The only difference was that cost was considered as the least important enabler for product developers. However, a recent study indicated that economic and environmental costs are significant factors in developing general-purpose LLMs [44].

Successful LLM development involves a complex interplay among technical innovation, regulatory compliance, production costs, and end-user needs. The aim should be to develop products that excel in functionality and positively impact the lives of those who rely on them without causing financial hardship. Thus, this study calls for collaboration between product developers, original equipment manufacturers, regulators, and other stakeholders to find solutions that align with technological advancements and societal expectations for affordability and accessibility.

Finally, the findings of this study were validated using MICMAC analysis, creating a graph that categorizes enablers based on their driving power and dependence power. In this graph, the enablers credibility, fairness, and accountability are in the first quadrant (autonomous) with low power, indicating that these variables are relatively independent and have limited influence on other variables. Usability and transparency are in the grey region between the third (linkage) and fourth (independent) quadrants with medium power, indicating a moderate influence on other variables and similarly influenced by them. Explainability falls in the grey region between the first (autonomous) and second (dependent) quadrants, also indicating a medium influence on other variables and a similar influence on them. Finally, cost falls under the fourth quadrant (independent), suggesting that it strongly influences other enablers without being significantly influenced by them. MICMAC analysis comprehensively explains the relationships and dynamics among variables within a complex system. This can help decision makers identify key drivers, dependencies, and interactions, enabling them to make informed strategic decisions and allocate resources effectively.

### Practical and Theoretical Implications

The study has one implication each for theory and for practice. For theory, this study extends the Fairness, Accountability, Transparency, and Explainability (FATE) framework [45] into a more comprehensive Cost, Usability, Credibility, Fairness, Accountability, Transparency, and Explainability (CUC-FATE) framework for developing LLMs for health care professionals. With respect to the implication for practice, this study is the first of its kind and provides a prescriptive framework for developing LLMs in health care, especially medical education. The findings of this study are useful for policy makers, medical device regulators, education policy makers, health care professionals, and product developers at the helm of creating Software as a Medical Device.

### Limitations

One of the limitations of the study is that the results largely rely on experts from India and the United Arab Emirates. Although technology and health care practices are standardized globally, the findings should only be generalized to the populations from these regions. This study provides insight into the relationships between different enablers but we did not further evaluate the strength of these associations. Graph theory or structured equation modeling can be used to address these gaps in future studies.

### Conclusion

This study emphasizes key factors for effective LLMs in medical education: credibility and accountability are vital enablers, while addressing bias and cost is crucial for enhancing LLM potential. Although important, transparency and explainability rank lower as LLM enablers among health professionals, suggesting a need for further education on this technology. Usability emerged as the least important factor; however, enhancing prompt use improves LLM accuracy. This study highlights a slight difference between product developers and end users. Although both groups prioritize credibility, fairness, accountability, transparency, and explainability, usability ranks lower for developers. Successful LLM development must balance innovation, compliance, costs, and user needs. Collaboration among stakeholders is crucial for aligning with technology and societal expectations.

## Abbreviations

AHP: analytical hierarchy process
AI: artificial intelligence
CI: consistency index
CR: consistency ratio
CUC-FATE: Cost, Usability, Credibility, Fairness, Accountability, Transparency, and Explainability
FATE: Fairness, Accountability, Transparency, and Explainability
LLM: large language model
MICMAC: cross-impact matrix multiplication applied to classification
NLP: natural language processing
RI: random index
TISM: total interpretive structural modeling

## Data Availability

The necessary data and calculations for the analytic hierarchy process model and the self-interaction matrix for the total interpretive structural model are available on a GitHub repository [46].
